# Dense neural networks for predicting chromatin conformation

**DOI:** 10.1186/s12859-018-2286-z

**Published:** 2018-10-11

**Authors:** Pau Farré, Alexandre Heurteau, Olivier Cuvier, Eldon Emberly

**Affiliations:** 1 Department of Physics, Simon Fraser University, 8888 University Dr., Burnaby, Canada; 2 Laboratoire de Biologie Moléculaire des Eucaryotes (LBME), CNRS, Bâtiment IBCG, Toulouse, 31062 France

**Keywords:** Chromatin folding, Dense neural network, HI-C, ChIP

## Abstract

**Background:**

DNA inside eukaryotic cells wraps around histones to form the 11nm chromatin fiber that can further fold into higher-order DNA loops, which may depend on the binding of architectural factors. Predicting how the DNA will fold given a distribution of bound factors, here viewed as a type of sequence, is currently an unsolved problem and several heterogeneous polymer models have shown that many features of the measured structure can be reproduced from simulations. However a model that determines the optimal connection between sequence and structure and that can rapidly assess the effects of varying either one is still lacking.

**Results:**

Here we train a dense neural network to solve for the local folding of chromatin, connecting structure, represented as a contact map, to a sequence of bound chromatin factors. The network includes a convolutional filter that compresses the large number of bound chromatin factors into a single 1D sequence representation that is optimized for predicting structure. We also train a network to solve the inverse problem, namely given only structural information in the form of a contact map, predict the likely sequence of chromatin states that generated it.

**Conclusions:**

By carrying out sensitivity analysis on both networks, we are able to highlight the importance of chromatin contexts and neighborhoods for regulating long-range contacts, along with critical alterations that affect contact formation. Our analysis shows that the networks have learned physical insights that are informative and intuitive about this complex polymer problem.

**Electronic supplementary material:**

The online version of this article (10.1186/s12859-018-2286-z) contains supplementary material, which is available to authorized users.

## Background

In eukaryotic cells, the condensation of the DNA into chromatin fibers that fold into specific 3D structures brings distant sites of the genome into spatial proximity. These conformations can modulate the expression of genetic information by altering the frequency of interaction between a distant regulatory element such as an enhancer, and the corresponding target gene promoter. The recent advent of high-throughput sequencing technology has allowed the genome-wide measurement of both chromatin structure via Hi-C contact maps [1, 2] as well as the bound locations of a great number of chromatin-associated factors through ChIP-seq methods [3, 4] and additional methodologies [5].

A large body of evidence supports the hypothesis that the spatial arrangement of bound chromatin factors along the DNA strongly influences the probability of chromatin contacts between distant genomic regions [6]. In particular, megabase-sized genomic compartments with similar chromatin states tend to interact with each other [1, 7]. At a finer sub-megabase scale, topologically associated domains [8–10] flanked by chromatin insulator or boundary elements [10–12] work as independent genomic units characterized by their self-interaction and repulsion with other genomic regions [13, 14]. Consequently, models that aim to predict how bound chromatin factors influence the folding of chromosomes are now being developed.

Most of the progress towards predicting chromatin conformation from the states of bound factors has come from simulating heterogeneous beads-on-a-string polymers whose bead types correspond to different chromatin states [15–28]. These simulations have been successful in corroborating that interactions between factors together with topological constraints may be responsible for driving chromatin conformation. Nevertheless, calculating the probability of contact between two genomic sites relies on sampling a vast number of polymer configurations. Consequently, exploring the conformational effects of altering the sequence of bound chromatin states is computationally challenging. An alternative Bayesian approach [29], has been successful in predicting the local contact maps from chromatin states without the need for polymer simulations and can rapidly calculate how contact probabilities change when chromatin states are altered. However, the chromatin states that were used as inputs were based on an unsupervised clustering of bound chromatin factors that did not take into account any structural information [30]. It is therefore unlikely that this classification constitutes the best 1D description of the sequence that determines chromatin structure and one may expect to achieve better predictive power by generating a conformation-specific annotation of the sequence of chromatin states. Methods that integrate both chromatin structure and sequence into a unified framework that can rapidly predict the respective contributions of changing sequence or structure are therefore needed.

Multi-layer neural networks that have been around for decades [31] provide the promise of a framework for learning the connections between the sequence of bound factors and chromatin structure. These networks consist of a series of units known as neurons that take in an input signal and have their weighted connections trained to reduce a defined cost function of the output. Many of the networks that are in use today for feature recognition are of a feed-forward structure consisting of a hierarchy of layers. These are dense neural networks (DNN) when they feature a high number of neurons in each layer and are considered to be “deep” when they feature a high number of layers. DNNs can also be coupled to trainable convolutional filters that help to discover important predictive features in the input that often reduce its dimensionality. Such networks are called convolutional neural networks (CNNs). The universal approximation theorem states that under mild assumptions, a feed-forward neural network with a single layer and a finite number of neurons can approximate any continuous function [32]. This capability is one of the main reasons of their great success at modeling complex problems with minimal design input by humans. Their use in bioinformatics has included predicting gene expression [33], the effect of sequence variants [34], predicting secondary structure [35], motif affinity [36] and filling in missing values in the human methylome [37]. In the context of Hi-C data analysis, multi-layer neural networks have been used to generate statistical confidence estimates for chromatin contacts [38] and to enhance the resolution of contact maps [39].

One challenge in using neural networks to model complex phenomenon is that they hide their inner workings behind large combinations of neuronal connections that are often hard to interpret. For this reason, DNNs tend to be seen as black boxes that can perform a great variety of tasks but offer little mechanistic explanation of how the inputs of the model are being used to generate the output. Nevertheless, in recent years a great amount of effort in the DNN community has been directed to developing techniques to infer how information is processed in these models ranging from sensitivity analysis to interpretability [40–46].

Here we apply dense neural networks (DNNs) to the problem of chromatin conformation. We show that using DNNs one is not only able to predict chromatin conformation from a sequence of DNA-bound chromatin associated factors, but also predict sequence from chromatin conformation. In addition, the model generates a biologically relevant 1D sequence annotation for chromatin states that is optimized to explain chromatin conformation. Furthermore, using sensitivity analysis we explore how the model relates sequence and conformation and unveil key regulatory features behind their connection. Such an approach highlights the importance of sequence neighborhood in structuring chromatin.

## Results and discussion

### Model predictions

**Fig. 1:**
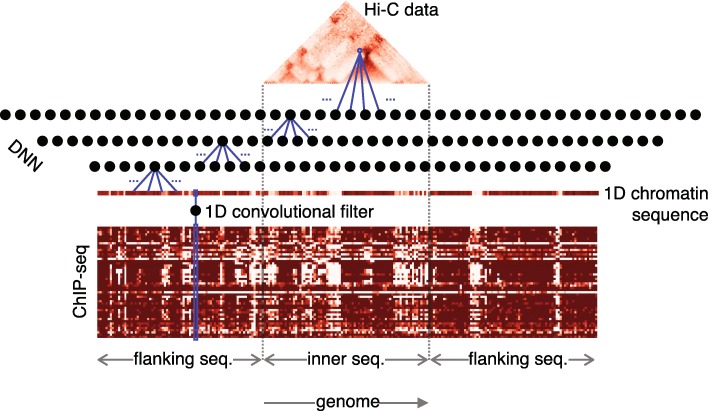
Schematic of the forward model convolutional neural network (CNN). This neural network is trained to predict chromatin contacts maps (Hi-C, top) from various chromatin-sequence factors (Chip-seq profiles, bottom). The Hi-C data to be predicted as an output is the upper diagonal of the Hi-C matrix of a *w*-wide genomic window. The input to the CNN is a 3*w*-long sequence that includes the *w*-long region of the Hi-C matrix (inner sequence) as well as two *w*-long sequences on each side (flanking sequences). The CNN is made of two parts. First, a sigmoid-activated convolutional layer reduces the *M* chromatin profiles to a 1D sequence profile. Then, the 1D sequence profile is fed to a ReLu-activated dense neural network (DNN) that predicts the Hi-C contact maps

As detailed in Methods, we developed multi-layer neural networks to predict chromatin conformation from sequence information in the form of the distribution of bound chromatin factors or vice versa, predict a likely sequence given only conformational data. Structure is represented as a contact matrix that gives how often every pair of sites of a fixed size/resolution in the genome were found to be in contact. We take as sequence data the enrichment of each site for a given bound protein that is associated with the folding of chromatin. (For the results that follow here, we use Hi-C data collected from *Drosophila Melanogaster* embryos at a resolution of 10 kbp for structure and we use the genome-wide distribution of 50 different bound chromatin-associated factors as sequence (see Methods).) Our aim is to train the neural networks to make predictions at the intra-chromosomal scale, i.e. using sequence data for a region of a chromosome predict the corresponding sub-region of the Hi-C contact map (or vice versa). To predict structure from sequence (what we will call the forward model), we couple a convolutional filter to a DNN (yielding a CNN) where the filter is used to compress the multitude of bound factors into a single 1D sequence that is then fed into the DNN to predict structure (see Fig. 1). This network architecture provides interpretability by giving a learned 1D sequence representation, {*σ*}, of the highly complex set of bound factors that best predicts structure, which the network calculates as the probability of contact, *P*(*c*_*ij*_) between all sites, *i* and *j* (see Methods for details). For predicting sequence from structure we use sub-regions of the Hi-C matrix and train the network to predict the corresponding region of the 1D sequence representation (see Methods). We call this the backward model (see Fig. 3a) and it requires a trained forward model to provide a 1D sequence representation that it can learn in going from structure to sequence. Once trained, the backward model can then predict a likely sequence representation from structure alone. We now provide details of how these models were trained and the biologically meaningful results that they generate.

**Fig. 2:**
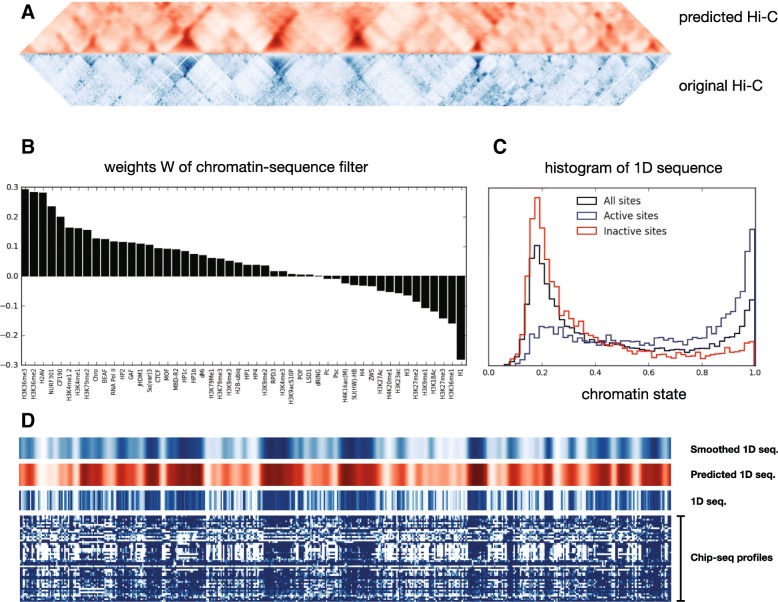
Structure and sequence predictions of forward and backward neural networks. **a** Distance-normalized Hi-C contacts predicted from the forward-model CNN in a 5 Mbp region of the test data set that was left out of the fitting procedure (chrom 3R 15–20 Mbp). A correlation of 0.68 between the original and predicted counts was obtained. **b** Weights of the sigmoid-activated convolutional filter applied to the chromatin factor sequences in order to generate the 1D sequence profile. **c** Histogram of the values of the 1D sequence obtained after the convolutional layer for all sites, transcriptionally active sites and transcriptionally inactive sites. **d** An independent DNN (backward model) was built to predict the 1D sequence from Hi-C data. From bottom to top, multiple chromatin factors were converted to this 1D sequence by running them through the convolutional filter of the forward model. Then, the backward model was used to predict those 1D sequences from Hi-C contacts. The predicted 1D sequence (red) and the original 1D sequence (below) showed a correlation of 0.73. (Top) A Gaussian-smoothed version of the original sequence showed a correlation of 0.93 with the predicted sequence from the backward model. The genomic region shown in (**d**) is the same 5 Mbp region of the test set shown in (**a**)

The forward model was fit using a training dataset along with a validation dataset that was used to check for convergence and to avoid over-fitting (see Methods). With the fitted forward model, a test set of sequences was then given as input, resulting in a set of predicted local contact maps. We found a Pearson correlation of 0.61 between the individual pairs of predicted and experimental contact maps in the test set. Nevertheless, since our data consists of overlapping sliding sequence windows (see Methods), a contacting pair is predicted multiple times as output from different sequences. This offers the opportunity of averaging the outputs from multiple windows and thus increase the predictive power of the model (akin to bootstrap aggregating, also known as bagging [47]). Upon doing so, the correlation between the entire predicted and experimental contact maps increased to 0.68 (Fig. 2a).

By training the forward model, the convolutional filter has been fit to optimize the predictive power of the high dimensional input sequence of bound factors to the contact map outputs. The learned weights in the convolutional filter, $W^{0}_{j}$ in Eq. 2, represent the strength of each chromatin factor in determining structure. Figure 2b shows the sorted distribution of these weights for all chromatin factors. The resulting distribution contains a significant amount of biologically relevant information. We find that one set of factors (negative weights) are associated with inactive/heterochromatic factors (H1, H3K27me3, H3K9me1), whereas the other set of factors (positive weights) corresponds to active/euchromatic factors associated with gene bodies (H3K36me2 and H3K36me3), promoters (RNA Polymerase II (RNAPII)), chromatin remodeler (NURF), poised enhancers (H3K4me1) or insulator proteins CP190, Chromator (Chro) and Beaf32 or CTCF, that are also associated with active genes [48, 49]. Interestingly, applying the filter to the input sequences and histogramming the resulting 1D chromatin annotation we find that it has a bimodal nature (Fig. 2c) where the inactive mode is bell-shaped around a value of ∼ 0.2, whereas the active mode is peaked at one. Moreover, histogramming the 1D chromatin state values of sites grouped by gene transcription level, we corroborate that small values (∼ 0.2) correspond to inactive chromatin whereas large values (∼ 1) correspond to active chromatin. This result is suggestive that experiments that ectopically activate or repress a gene could be used to test the structural changes predicted by our model due to “flipping” the state of the corresponding site from inactive to active, or vice-versa. Similarly, grouping sites based on the “chromatin colors” classification [30] we also find that small annotation values correspond to heterochromatic classes while large values correspond to euchromatic ones (see Additional file 1: Figure S1). Thus the fitted filter has naturally grouped the multitude of different chromatin factors into two groupings, with the heterochromatic factors playing a more dominant singular role in shaping structure and the euchromatic factors having a more heterogeneous influence.

**Fig. 3:**
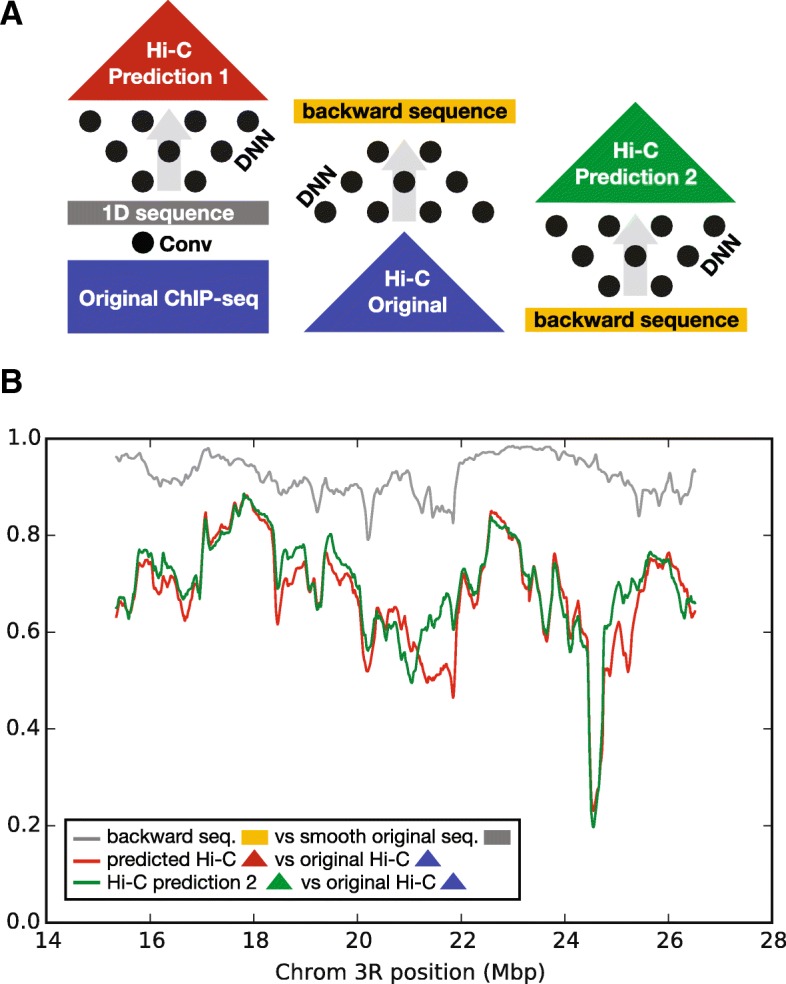
Detecting potential discrepancies between sequential and structural datasets. **a** Schematic of the three sequence-structure predictions performed. On the left, the original ChIP-seq is fed to a CNN (forward model) and outputs both a 1D sequence and a predicted Hi-C. In the centre, the original Hi-C is fed to a DNN (backward model) to predict the 1D sequence found in the forward model called the backward sequence. On the right, the backward sequence is fed to the dense neural network of the forward model (without fitting it again) to generate a new Hi-C prediction (Hi-C prediction 2), based on the backward sequence derived from the original Hi-C. **b** Shown is the correlation between the data generated by the models in (**a**) along the *w*-wide genomic windows. Genomic regions where the backward sequence differs from the original sequence tend to coincide with regions where the predicted Hi-C and the original Hi-C differ (0.35 correlation). There is an improvement between the predicted Hi-C and the original Hi-C when using the backward predicted sequence as input

We solved the inverse problem (structure to sequence) by fitting an independent multi-layer DNN (see Methods). This model, named the backward model, predicts the 1D chromatin sequence annotation from the forward model using just the local Hi-C contact map as input. The backward model thus predicts a likely sequence of chromatin states that could form that map. Applying the trained backward model to the test set, the individual sequences predicted from the local contact maps had a correlation of 0.66 with the original 1D sequence and a correlation of 0.73 after averaging overlapping windows (Fig. 2d). The predicted profiles visually resemble a smoothened version of the original 1D sequence from the convolutional filter. This was corroborated by performing a Gaussian smoothing on the original 1D sequence, with the resulting smoothed sequence now having a correlation of 0.93 with the backward model prediction (Fig. 2d). Based on this finding, we hypothesize that the forward-model may in fact be doing smoothing internally in the DNN by predicting chromatin contacts based on a local average of the sequence. We tested this by feeding the Gaussian-smoothened 1D sequence from the convolutional filter as input into the DNN layers of the forward model. The predicted contacts from the smoothed sequence showed a correlation of 0.98 with the previously predicted contacts derived from the non-smoothed sequence (shown in Fig. 3b), indicating that the forward-model network generates a similar contact map output from a smoother description of chromatin factors.

Next, we inspected how the predictions obtained from the forward and backward models compare to each other. We thus looked at the correlation between the forward 1D sequence (derived from ChIP-Seq) and the backward 1D sequence (derived from contacts) in the test set (Fig. 3b). We found that regions where these sequences differed most were regions where the correlation between the predicted Hi-C counts from the forward and original counts tended to also be poor (0.35 correlation between the two trends). These concordant discrepancies between the forward and the backward model predictions could be indicative of divergencies between the actual state of the cells used for measuring sequence and chromatin contact maps. We further tested this hypothesis by feeding the sequences predicted from the backward model as input to the DNN of the forward-model, obtaining a new set of predicted contact maps (see schematic in Fig. 3a). We find that the correlation between the predicted and original contact maps improves from 0.68 to 0.71 using the sequences from the backward model compared to the sequence from the convolutional filter applied to the ChIP-seq profiles (Fig. 3b). This thus indicates that the observed discrepancies are generally not a result of noisy sequence prediction, instead they are suggestive of small changes in chromatin sequence that generally improve structure prediction. In addition, genomic regions where chromatin structure is consistently poorly predicted (eg. the large dip the correlation around ∼ 24−25 Mbp in Fig. 3b) may be indicative that the principles of chromatin folding learned by our models are are not the primary drivers of conformation in these locations. Our findings thus highlight two aspects: First, 2D contact maps can be efficiently encoded into a 1D vector and decoded back (our backward and forward model effectively work as an auto-encoder, similarly to [39], with the difference that our embedded feature vector is the chromatin sequence). Second, a backward model that predicts sequence from structure can be used to identify locations in the genome where sequential and structural datasets likely differ from one other. One could imagine this to be a powerful technique to analyze phenotype-to-genotype linkages by identifying regions where chromatin states are likely to vary by using contact maps from cells of differing tissue, developmental time or disease.

### Spatial analysis of conformational effects

In this section we focus on determining what characteristics of the chromatin sequence influence contact maps the most, and vice-versa, what elements of a local contact map are important for inferring sequence. The following analysis thus serves a double purpose: On the one hand, it answers specific questions about chromatin folding. For instance, it quantifies how a particular structure may be altered by making a given genomic site more active or inactive. Secondly, it verifies that the predictions from our neural networks come from a correct representation of the underlying biological mechanisms rather than the exploitation of data artifacts.

**Fig. 4:**
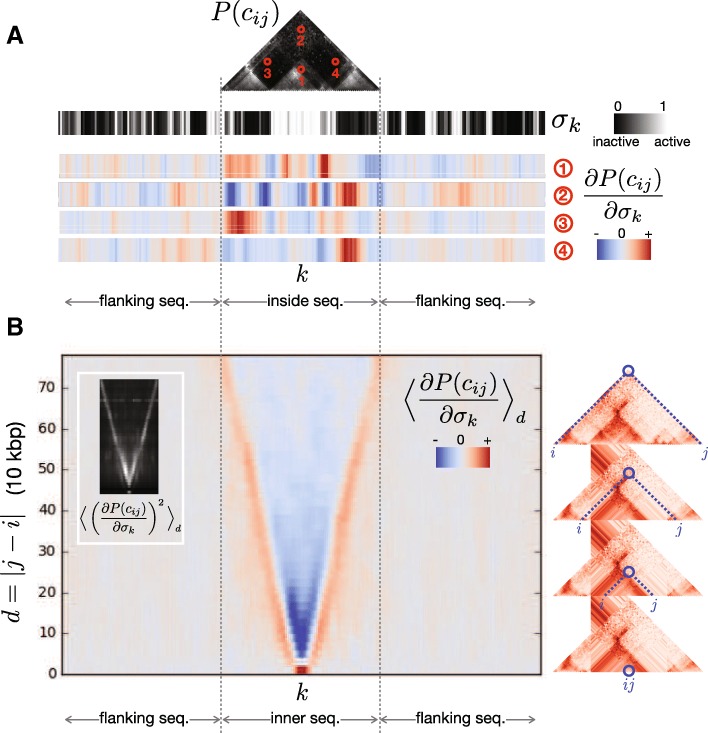
Gradient analysis of probabilities of contact. **a** The gradient of the probabilities of contact with respect to the surrounding chromatin states was evaluated at every genomic region. As an example, we show a contact map *P*(*c*_*ij*_) together with its respective chromatin states *σ*_*k*_ (chromosome 2L 9.15–11.55 Mbp). The gradient was evaluated in four different pairs of sites *i* and *j* marked as red circles, and it shows how chromatin states should be altered in order to increase the probability of contact between those sites. The gradient profiles thus suggests the activation or inactivation of regions depending on the location of *i* and *j* in the contact map and the sequence of chromatin states. **b** Genome-wide average of probability gradients *∂P*(*c*_*ij*_)/*∂σ*_*k*_ sorted by distance of contact between *i* and *j*. An increase of inactive states between pairs of sites typically increases the probability of contact, especially at shorter distances of contact (*d*<300 kbp). An increase of active states on the sites of contact itself typically increases the probability of contact between sites. The inset shows the average squared gradients, which are indicative of the average magnitude of the gradients, therefore highlighting the chromatin regions with the largest weight on determining probability of contact

First, using the forward model, we measured how sensitive the probability of contact *P*(*c*_*ij*_) is to sequence *σ*_*k*_ by calculating the gradient of the probability of contact of a pair of sites *∂P*(*c*_*ij*_)/*∂σ*_*k*_ at each site *k* of the 3*w*-long input sequence. This quantity can be obtained by the method of back-propagation (see Methods) and highlights how the probability of contact would change upon increasing the value of *σ*_*k*_ (i.e. making the chromatin more active at that location *k*). Alternatively, gradient values can also be interpreted as how chromatin states must be altered in order to increase the probability of contact. A negative value of the gradient would imply that to increase the contact probability one would have to decrease the value of *σ*_*k*_, making the state more inactive. Therefore, such analysis highlights the conformational effect that would be expected upon mutating/altering the bound sequence at each particular location of the genome (Fig. 4a).

To examine the general effects of how varying the sequence affects the probability of contact we averaged *∂P*(*c*_*ij*_)/*∂σ*_*k*_ over the test data. Specifically, for each pair of sites *i* and *j* in the genome separated by a distance *d*=|*j*−*i*|, we calculated *∂P*(*c*_*ij*_)/*∂σ*_*k*_ for the data in which (*i*,*j*) appear centred in the sequence *w* (for an example, see the pairs of sites (1) and (2) in Fig. 4a). Then we averaged all pairs of sites in the test data that meet these criteria, obtaining a sequence of average gradients for centred sites situated at a distance *d*. In Fig. 4b we show the 〈*∂P*(*c*_*ij*_)/*∂σ*_*k*_〉_*d*_ at each distance *d* as a function of relative position *k*. We find that making the regions between contacting sites more inactive tends to favor more contact (〈*∂P*(*c*_*ij*_)/*∂σ*_*k*_〉_*d*_<0). This is strongest at shorter distances of contact (*d*<300 kbp) and becomes weaker as the distance increases. On the other hand, making the sites of contact more active also increases the contact probability (〈*∂P*(*c*_*ij*_)/*∂σ*_*k*_〉_*d*_>0), regardless of distance of contact. The heat map also shows that active chromatin immediately outside *i* and *j* at shorter ranges of contact can also increase the probability of contact.

The gradient squared (*∂P*(*c*_*ij*_)/*∂σ*_*k*_)^2^ highlights where the magnitude of the gradient is the greatest, and indicates which sequence locations dominate contact probabilities. In the inset of Fig. 4b we observe that, overall, the chromatin states at the sites of contact are the strongest determinants of contact probability. Nevertheless, the contact probability of sites situated at a shorter distance (*d*<300 kbp) are also strongly determined by the state of the chromatin neighbours in between and outside the sites.

**Fig. 5:**
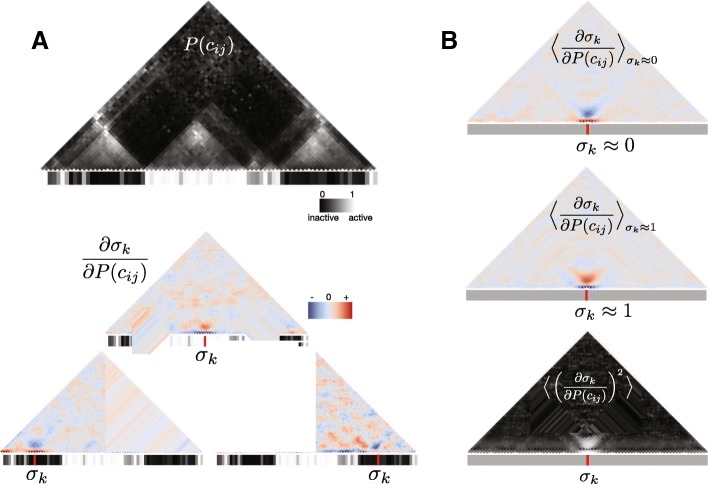
Gradient analysis of probabilities of chromatin states. Gradient analysis of probabilities of chromatin states. **a** At the top, a contact map and inner sequence of a genomic window used as an example to evaluate gradients (chromosome 2L 9.95–10.75 Mbp). At the bottom, the gradient of the chromatin states of three different locations *σ*_*k*_ of the sequence (marked as red) with respect to the probabilities of contact (in the same genomic window as above). The heat maps indicate how the probabilities of contact *P*(*c*_*ij*_) would need to change in order to increase value of the chromatin state *σ*_*k*_ (make it more active), or equivalently, how *σ*_*k*_ would change when increasing *P*(*c*_*ij*_). **b** Genome-average of gradients for subsets of sites where the central chromatin state is either inactive (*σ*_*k*_≈0) or active (*σ*_*k*_≈1), and genome-average gradients of the central chromatin state. On average, when *σ*_*k*_ is an inactive state, an increase of contacts between the sites surrounding *σ*_*k*_ makes *σ*_*k*_ more inactive (negative gradient). For the active state, an increase of contact probabilities between sites surrounding *σ*_*k*_ tends to make the sites more active (positive gradient). The average gradient square highlights that the contacts that are most informative about the chromatin state at a given location are the contacts between the sites that flank the location

Second, we performed similar gradient analysis on the backward model that predicts chromatin states from contact maps. For the backward model the gradient corresponds to *∂σ*_*k*_/*∂P*(*c*_*ij*_), and indicates how a change in contact between two sites *i* and *j* would be reflective of a change in the chromatin state at site *k*. We evaluated *∂σ*_*k*_/*∂P*(*c*_*ij*_) at each genomic location and it can be visualized as a map of gradients with the same size as the contact map. In Fig. 5a we show at the top the contact map and sequence of a particular genomic location. At the bottom of Fig. 5a, we evaluate the gradient *∂σ*_*k*_/*∂P*(*c*_*ij*_) for different *σ*_*k*_ positions in the same genomic location. The heat maps indicate how the chromatin state *σ*_*k*_ would change when increasing a particular *P*(*c*_*ij*_). In Fig. 5b, we averaged over the test set the gradients for those *w*-long sequences whose central chromatin state was either inactive or active. This calculation highlights that when the chromatin state is inactive, an increase in contact between sites situated at the left and the right of the inactive state would be indicative of the inactive state becoming even less active (or more inactive). The opposite trend is held for active sites, with an increase of contact between neighbouring sites corresponding to the site being more active. Last, by looking at the average squared gradient we find that the contacts between sites on the left and right of the site of interest are the main determinants of the chromatin state at that site.

## Conclusion

In this paper we have presented a method using dense neural networks (DNN) for predicting chromatin contact maps from sequences bound by chromatin factors and vice versa. Notably, although a certain amount of human-guided design choices went into structuring the DNN, a large part of the model selection behind it was done automatically. By fitting a convolutional filter, we were able to reduce the high dimensional input of multiple chromatin factors to a single 1D chromatin state sequence that was most predictive of structure. Furthermore, by building an inverse model that predicts the chromatin state sequence from contact maps, we could show that the 2D contact maps can effectively be compressed into a 1D sequence and decompressed back. This supports the theory that chromatin folding (for scales at least < 800 kbp) is strongly determined by chromatin states arising from a sequence of bound factors.

By analyzing how varying the inputs to the two neural networks (forward and backward models) changed the outputs, we highlighted that chromatin conformation is a non-local problem; the probabilities of contact between each pair of sites depends on the chromatin states in the larger neighbourhood. In general, the presence of inactive chromatin between two contacting sites or active chromatin on the sites themselves and outside their flanks increases the probability of contact. Nevertheless, the probability of contact between sites situated at a larger distance (> 300 kbp) is largely determined by the chromatin states of the contacting sites themselves.

The work presented in this paper is a proof-of-concept that can easily be extended to capture more biological features of interest. For instance, one could use a larger number of convolutional filters, which would provide a richer biological description of chromatin states (i.e. not just a simple ’inactive’ versus ’active’ state reduction as was done here). This could also include going to finer resolutions both at the level of bound factors and the contact map [12, 50], and there is no reason why they have to be at the same spatial scale. One could also introduce additional types of sequence as inputs, such as genomic annotations, gene expression measurements, genomic mapability and other relevant information to the problem. In addition, one may also be able to introduce non-sequential inputs, that may allow the inclusion of experimental details such as developmental times, cell types or temperatures, and thus allow the modeling of a heterogeneous mixture of cells.

## Methods

### Hi-C data

Chromatin structural information comes in the form of genome-wide contact maps that were obtained from the publicly available Hi-C experiments done by Schuettengruber et al. [51] (GSE61471), performed on 3000-4000 *Drosophila melanogaster* embryos, 16-18 hours after egg laying. The contact map is an array whose elements *n*_*ij*_ are the number of times a particular pair of genomic sites were found to be in contact. The contact map was built using a set of *N* non-overlapping sites of a fixed size (we used a size of 10 kbp which gave *N*=9663 for all the autosomal chromosomes of *D. melanogaster*). The counts, *n*_*ij*_, between all pair of sites *i* and *j* in the contact map were determined by counting up all sequenced pairs from the Hi-C measurement that fell into a given pair yielding an *N*×*N* symmetric matrix (and each unique sequence pair was only counted once to reduce experimental bias, as suggested in [8]).

We normalized the contact map using the ICE method [52] so that total number of counts along each row across the contact map was the same. Then, we measured the average number of contacts at each distance of contact and divided the Hi-C counts by it. This correction removed the strong decaying signal as a function of the distance between contacting sites due to the entropic polymer effect (such as done in [53]). Our final contact maps, which we label as *P*(*c*_*ij*_), correspond to contact enrichments at a given distance and are proportional to the actual probabilities of contact when the polymer entropy is removed.

### ChIP-seq data

For the sequence of bound factors, we used the enriched genomic regions of 50 chromatin factors measured with ChIP-seq in 14-16 hour *D. melanogaster* embryos [54]. Specifically, we downloaded the following factors: BEAF, H3K23ac, H3K79Me1, HP1, POF, CP190, H3K27Ac, H3K79me2, HP1b, Pc, CTCF, H3K27me2, H3K79me3, HP1c, Psc, Chro, H3K27me3, H3K9acS10P, HP2, RNA Pol II, GAF, H3K36me1, H3K9me1, HP4, RPD3, H1, H3K36me2, H3K9me2, JHDM1, SU(HW)-HB, H2AV, H3K36me3, H3K9me3, LSD1, Su(var)3, H2B-ubiq, H3K4me1, H4, MBD-R2, ZW5, H3, H3K4me1, H4K16ac(M), MOF, dMi, H3K18Ac, H3K4me3, H4K20me1, NURF301, dRING. This data is publicly available at http://www.modencode.org/ as part of the modENCODE project.

Using the same genomic binning that was used in constructing the contact map (size of 10 kbp), we built *M* (here *M*=50) sequence profiles of length *N* (here *N*=9663) by calculating what fraction of each site was enriched for a given chromatin factor. Therefore, the values of the sequence profiles range from 0 (factor is not present) to 1 (the bin is fully occupied by the factor).

### Transcription data

Gene transcription data was obtained from publicly available RNA tag sequences detected with Illumina GAII with the digital gene expression (DGE) module from duplicate RNA samples from Kc167 cells [30](GSE22069).

We assigned a transcription score to each genomic bin by multiplying RNA counts by the fraction of the genomic bin that is occupied by the the gene in question. Next, we classified each bin into either active of inactive by tresholding transcription scores at one (inactive: transcription score < 1, active: transcription score > 1).

### Dense neural networks for connecting conformation to sequence

A schematic for our model that uses a convolutional neural network (CNN) to predict chromatin contact maps from bound-DNA sequence data is shown in Fig. 1. The output of the network is a local contact map of size *w*×*w* that contains *w*(*w*+1)/2 independent elements (we take *w*=80 that gives 3240 network outputs). With respect to input, based on our prior work [29] that showed the importance of flanking sequence neighborhoods, we take the sub-sequence of length *w* from the *M* sequence profiles that is centered on the *w* sites of the contact map, along with flanking sequences of size *w* giving an input array of size *M*×3*w*.

Our CNN for predicting chromatin conformation from sequence, which will be referred as “the forward model”, has interpretability in mind. First, a convolutional filter with width equal to one and an sigmoidal output function acts on the (*M*×3*w*) input reducing its dimensionality to a one-dimensional 3*w*-long vector (Fig. 1) whose individual values range from 0 to 1. This vector can be interpreted as a one-dimensional sequence of chromatin states (with values between 0 and 1) that is used as input to the rest of the neural network to predict contact maps. More specifically, if we denote by $\vec {x}_{i}$ the *i*th position of the input sequence, with dimension *M* equal to the number of chromatin factors, the value of the 1D chromatin annotation at that position, *σ*_*i*_, is obtained from

1 $${} \sigma_{i} = \frac{e^{E_{i}}}{1+e^{E_{i}}},  $$

with

2 $$ E_{i} = \sum\limits_{j}^{M} W^{0}_{j}\cdot x_{i,j}+\beta_{0},  $$

where $W^{0}_{j}$ and *β*_0_ are the trainable weights of the convolutional filter. The index *j* corresponds to each of the *M* chromatin factors. The fitted filter thus denotes the weights applied to each of the *M* bound factors for classifying the chromatin into a single sequence that is the best predictor of structure.

Next, the resulting 1D sequence profile of size 3*w* is fed to a DNN with multiple layers of increasing size, where the last layer has *w*×(*w*+1)/2 outputs corresponding to the values of a local contact map for *w* bins. The value obtained at neuron *i* of layer *n*, $y^{n}_{i}$, is calculated using the values of all neurons *k* in the previous layer $y^{n-1}_{k}$,

3 $$ y^{n}_{i} = ReLu \left(\sum\limits_{k} W^{n}_{k} \cdot y^{n-1}_{k} + \beta_{i} \right),  $$

where $W^{n}_{k}$ is a matrix of weights applied to each neuron of the previous layer, *β*_*i*_ is a constant, and *ReLu* is the rectified linear unit function, namely *f*(*x*)=max(0,*x*), which helps to introduce non-linearities and sparse activation (50*%* of neurons are activated) while remaining easily computed and differentiated [55]. Both $W^{n}_{k}$ and *β*_*i*_ are trainable parameters.

The cost function to be minimized during the fitting procedure was taken to be the mean squared error between experimental and predicted distance-normalized contact maps, along with L2 regularization of the filter weights. The technique of dropout regularization, that consists on setting the output of randomly selected neurons to zero with a given probability was used to control for over-fitting [56]. Optimization was done using stochastic gradient descent. We use the Python package Keras (https://github.com/keras-team/keras) to code our model, running on top of TensorFlow (https://www.tensorflow.org/).

For the particular example used in the Results the following network was built and fit. The output from the convolutional filter is fed to four ReLu-activated layers of exponentially increasing size, where the last layer is the output layer with same size as the output chromatin map data (Layer 1: 460 neurons, Layer 2: 881 neurons, Layer 3: 1690 neurons, Output Layer: 3240 neurons). A dropout of 0.1 was applied to the dense layers during training. The training was divided into 30 batches, only evaluating the cost function on one batch at a time. At the end of each epoch an independent validation set was used to evaluate the cost function independently to avoid over-fitting. The fitting procedure ended when the cost function calculated in the validation set converged. Results were then calculated on the test data set. The training converged in approximately 30 minutes on a personal laptop.

In addition, we also built a dense neural network (DNN) that solves the inverse problem. Namely, it is trained to predict the previously found 1D chromatin annotation from contact maps alone. The architecture of this network resembles an inverted version of the forward-model, and we thus name it “the backward model”. This network outputs a *w*-long vector of 1D chromatin states from the *w*×(*w*+1)/2 contacts between pairs of sites in the sequence window. Note that the output of the backward model is *w*-long in contrast to the 3*w*-long sequence used as input of the forward model. This is because the flanking regions are hard to predict using just the contact map from the *w*-long interior region, and trying to predict the 3*w* long sequence leads to convergence errors in the procedure. The network is comprised of multiple ReLu-activated dense layers, except for the last output layer which is sigmoid-activated. The “backward model” presented in Results was made of three layers of exponentially decreasing size, the first two ReLu-activated, and the last layer (output layer) is sigmoid-activated (Layer 1: 943 neurons, Layer 2: 274 neurons, Output Layer: 80 neurons). It was fit using the same procedure as the feed-forward network.

### Datasets for training, validating and testing

For training and validating the models, we have used sequence and contact map data from the *D. melanogaster* chromosomes 2L, 2R, 3L and the first half of chromosome 3R (from 1 to 12.95 Mbp). From these regions we obtained 13814 pairs of local sequences and structures using *w*=80. We randomly subsampled 80% of this data as a training set (11052 pairs) and 20% was set aside as a validation set that was not used in the parameter fitting procedure (2768 pairs). For testing the predictions of the model, we used the remaining second half of chromosome 3R (from 14.95 to 26.91 Mbp) as a test set, which contained 2112 pairs of sequences and structures. (It should be noted that our datasets included left-right inverted versions of the data, as the directionality of the genome should not influence the relationship between chromatin contacts and sequence. This thus allowed us to build a dataset of sequences and structures with a size approximately twice the length of the binned genome.)

### Gradient analysis of DNNs

We calculated gradients of the network models using a method known as sensitivity analysis [57, 58]. In particular, we followed the DeepTaylor tutorial in www.heatmapping.org to calculate for a given output neuron the gradient of the output function with respect to the input variables. First, we exported the values of every layer of our trained neural network to text files. Then, for each neuron in the last layer that we wanted to calculate the gradient of, we rebuilt the trained neural network and only included the neuron of interest in the last layer. These neural networks were built using a minimal neural network implementation that can be found in the script “modules.py” from www.heatmapping.org/tutorial. The back-propagation of the gradient of the neuron in the last layer with respect to the input data was done using the methods in “utils.py” from www.heatmapping.org/tutorial as described in the website tutorial.

## Additional file

Additional file 1 **Supplementary EPS figure**. Correlation between 1D sequence annotation and chromatin colors. Histogram of values of the 1D sequence obtained after applying the convolutional layer. Shown are all sites, as well as sites in which more than 50% of their 10kb-long genomic bin belonging to one of the the five chromatin-color states classified by Fillion et al. Cell 143, 212-224 (2010). (EPS 369 kb)

## Abbreviations

CNN: Convolutional neural network
DNN: Dense neural network
